# Relationships of walking activity with depressed mood and suicidal ideation among the middle-aged Korean population: a nationwide cross-sectional study

**DOI:** 10.3389/fpsyt.2023.1202068

**Published:** 2023-09-05

**Authors:** Yujin Ko, Yong Hyuk Cho, Gi Woon Kim, Chang Hyung Hong, Sang Joon Son, Hyun Woong Roh, Jieun Moon, Sangsoo Han

**Affiliations:** ^1^Department of Psychiatry, Gangnam Severance Hospital, Yonsei University College of Medicine, Seoul, Republic of Korea; ^2^Department of Psychiatry, Ajou University School of Medicine, Suwon, Republic of Korea; ^3^Department of Medical Sciences, Graduate School of Ajou University, Suwon, Republic of Korea; ^4^Department of Emergency Medicine, Soonchunhyang University Bucheon Hospital, Bucheon, Republic of Korea; ^5^Department of Biostatistics, Clinical Trial Center, Soonchunhyang University Bucheon Hospital, Bucheon, Republic of Korea

**Keywords:** walking, exercise, suicidal ideation, depressed mood, nationwide, cross-sectional study

## Abstract

**Introduction:**

The suicide rate of middle-aged adults has increased rapidly, which is a significant public health concern. A depressed mood and suicidal ideation are significant risk factors for suicide, and non-pharmacological interventions such as exercise therapy have been suggested as potential treatments. Walking is a feasible and accessible form of exercise therapy for middle-aged adults.

**Methods:**

We conducted a study based on the Seventh Korea National Health and Nutrition Examination Survey (2016–2018) data of 6,886 general middle-aged adults in South Korea to investigate the relationships of walking exercise with depressed mood and suicidal ideation. Multiple logistic regression analysis was used to adjust for confounding variables. Sampling weights were applied to obtain estimates for the general Korean population.

**Results:**

Participants who walked ≥5 days per week had a significantly lower odds ratio (OR) for depressed mood [OR = 0.625, 95% confidence interval (CI): 0.424–0.921, *p* = 0.018] and suicidal ideation (OR = 0.252, 95% CI: 0.125–0.507, *p* < 0.001) compared to those who never walked, regardless of the duration of exercise. The same results were obtained for males after stratifying the data by sex and suicidal ideation was associated with walking in females.

**Conclusion:**

Regular walking exercise was associated with diminished mental health problems in middle-aged adults. Light walks may serve as a useful starting point for patients with serious mental health issues, such as suicidal ideation.

## Introduction

Suicide is a leading cause of death worldwide, accounting for more than 700,000 deaths annually (1). A large amount of resources is being invested in suicide prevention and many approaches are being studied. Among the OECD countries, South Korea has had the highest suicide rate, and the government has been researching policies to address this issue (2). However, suicide rates remain high. Suicide is an important public health issue in all age groups, but the severity of the problem is particularly increasing in the middle-aged population. The suicide rate of middle-aged adults in the United States has increased by >40% over the past 20 years, whereas there has been little change in other age groups (3). A similar trend in suicide rates has also been observed in South Korea (4). Middle age is a crucial period in terms of professional career and an important point of transition from youth to older age. Thus, the increasing suicide rate among middle-aged adults is an important public health issue.

Many studies have focused on identifying the risk factors for suicide with the goal of facilitating prevention. Depression has been extensively researched as a risk factor contributing to suicidal ideation and behavior, and is consistently included in suicide risk assessments and guidelines (5, 6). Suicidal behavior is a symptom of depression, and depression is the most common mental illness in patients who exhibit suicidal behavior (7). Accordingly, research is being conducted on pharmacological and non-pharmacological treatments for managing depression, as a risk factor for suicidal behavior, and for managing suicidal ideation, which is directly associated with suicidal attempts/death (8). Exercise is an alternative treatment option (9, 10) but regular and intense exercise therapy is burdensome for most patients. Walking is a form of exercise that imposes a smaller burden on patients. Growing evidence suggests beneficial effects of walking on mental health conditions such as depression, anxiety, and psychological stress (11). However, information regarding the duration of walking that provides beneficial effects is lacking. A study on the association between walking and suicidal ideation was limited to a subset of cancer patients (12). Additionally, no results applicable to middle-aged adults were reported.

The current study aimed to examine the associations of walking exercise with depressed mood and suicidal ideation in middle-aged individuals in South Korea. We also investigated whether the effects of walking on depressed mood and suicidal ideation vary by walking duration. To our knowledge, this is the first study to examine the effects of walking on depressed mood and suicidal ideation in a general middle-aged population.

## Methods

### Study design and participants

In this nationwide cross-sectional study, information was collected from the Korean National Health and Nutrition Examination Survey (KNHANES), which is a survey that has been carried out by the Korean Centers for Disease Control and Prevention (KCDC) since 1998 (13). The KNHANES is a national cross-sectional survey that obtains information on health behavior *via* interviews, self-report measures, a physical examination, and a nutritional survey. A stratified, multistage probability sampling design was employed using household registries; sampling units were based on geographical area, sex, and age. Approximately 10,000 independent samples were obtained annually from 192 primary sampling units PSUs. Each PSU comprises Korean residential addresses, which are included based on the administrative district and housing type.

This study concentrated on participants aged 40–60 years who participated in the Seventh KNHANES (2016–2018). The exclusion criteria were failure to complete the depressed mood and suicidal ideation questionnaire or walking activity questionnaire.

### Definition of walking activity

We defined walking activity in our study as walking for >10 min continuously per week, using the criteria from the International Physical Activity Questionnaire-short form (IPAQ) (14). We tried to find out the effect of the exercise more clearly by setting the minimum walking time as the criterion. Also, a supplementary analysis was conducted for 30 min of walking (15). Participants were asked to report how many days per week they engaged in walking, including commuting, going to school, and during exercise. The response options were as follows (1): no walking (2), 1–2 days per week (3), 3–4 days per week, and (4) ≥ 5 days per week (16, 17). Participants who reported walking were then asked to indicate the typical duration thereof. Then, we calculated the total weekly walking duration by multiplying the walking activity per week by the duration of each walking occasion.

### Definitions of depressed mood and suicidal ideation

To evaluate depressed mood, participants were asked if they had consistently felt sadness or despair that interfered their everyday life for ≥2 weeks in the past year. The response options were “yes” and “no” (18). Suicidal ideation was assessed by asking participants if they had seriously considered suicide in the past year; the response options were again “yes” and “no” (19).

### Potential mediators and confounders

Demographic characteristics, socioeconomic status, medical history, and lifestyle habits data collected during the health interview and physical examination were analyzed. Body mass index (BMI) was calculated by dividing weight by height squared. All participants were categorized as nonsmokers, ex-smokers, or current smokers. Alcohol consumption was classified as none, ≤ 1 drink/month, 2 drinks/month to 3 drinks/week, or ≥ 4 drinks/week. Education level was categorized as elementary school, middle school, high school, or college/university. Occupation was classified as unemployed, simple labor (e.g., technician or low-level laborer), agriculture, fisheries, sales and services, or office worker (20). Household income level was divided into quartiles. Marital status was categorized as single, married, or separated/divorced/widowed. Sleep duration was obtained by asking the participants “How many hours do you sleep each day?” The participants were also evaluated for the presence of major comorbidities including hypertension, diabetes, dyslipidemia, stroke, ischemic heart disease (myocardial infarction, and angina), asthma, and malignant tumors (lung, stomach, liver, colon, breast, uterine, or cervical).

### Statistical analysis

The chi-square test was used to compare categorical variables, while Student’s *t*-test was used to compare continuous variables. We used logistic regression analysis to calculate the odds ratios (ORs) and 95% confidence intervals (CIs). We adjusted for explanatory variables including age, sex, smoking status, alcohol consumption, educational level, household income, marital status, occupation, sleep duration, and comorbidities that can affect depressed mood and suicidal ideation. Subgroup analysis was performed according to sex (male or female). As the KNHANES data comprises complex survey data, complex sample analysis was performed. Population weights were applied to ensure that the data were representative of the entire Korean population. The statistical analysis was carried out using IBM SPSS Statistics for Windows software (version 26.0; IBM Corp., Armonk, NY, United States). A two-tailed value of *p* <0.05 was considered significant.

### Ethics statement

The Institutional Review Board of the KCDC approved the KNHANES VII (approval no. 2018-01-03-P-A), and all participants provided informed consent. This study followed the principles of the Declaration of Helsinki and complied with relevant guidelines and regulations.

## Results

**Figure 1:**
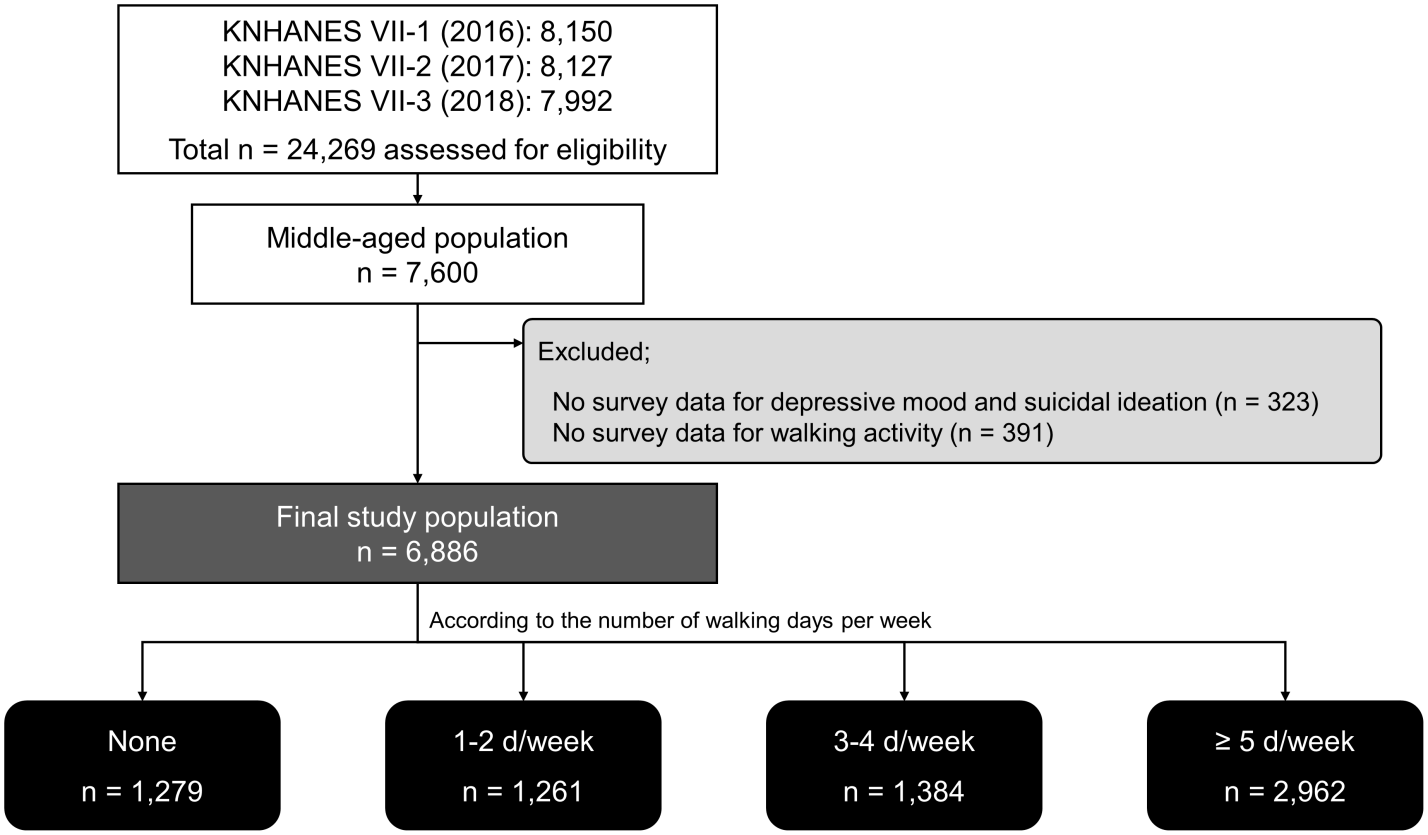
Flow chart of the study. KNHANES, Korea National Health and Nutrition Examination Survey.

In total, 24,269 people participated in the KNHANES during the study period, including 7,600 middle-aged participants (40–60 years old). After excluding people who did not complete the depressed mood and suicidal ideation survey (323 people) or walking activity survey (391 people), we analyzed the data of 6,886 people (Figure 1).

### Baseline characteristics of the participants according to the number of walking days

The 6,886 participants were divided into those who never walked (1,279; 18.6%), those who walked 1–2 times per week (1,261; 18.3%), those who walked 3–4 times per week (1,384; 20.1%), and those who walked >5 times per week (2,962; 43%). The mean walking durations per session of these four groups were 0, 56.3, 53.6, and 66.2 min, and the mean weekly walking durations were 0, 144.2, 234.7, and 491.1 min, respectively. The participants’ demographic and characteristics are listed in Table 1. Sex, smoking status, education level, occupation, household income, marital status, sleep duration, and hypertension differed significantly among the four groups. The depressed mood rates also differed significantly among the groups; depressed mood was reported by 60 (4.7%), 37 (2.9%), 38 (2.8%), and 93 (3.1%) participants who never walked, walked on 1–2 times days week, walked on 3–4 days per week, and walked on >5 days per week, respectively (*p* = 0.02). The number of participants with suicidal ideation also differed significantly among the four groups [29 (2.3%), 17 (1.4%), 13 (0.9%), and 23 (0.8%), respectively, *p* < 0.001]. The baseline characteristics after population weighting is also presented in Supplementary Table S1.

**Table 1:** Baseline characteristics according to the number of walking days per week.

	None	1–2 d/wk	3–4 d/wk	≥ 5 d/wk	*p*-value
	(*n* = 1,279)	(*n* = 1,261)	(*n* = 1,384)	(*n* = 2,962)	
Age, years	50.47 ± 6.04	49.86 ± 6.05	50.17 ± 5.99	50.25 ± 6.17	0.077
Sex, *n* (%)					<0.001
Male	634 (49.57)	565 (44.81)	554 (40.03)	1,171 (39.53)	
Female	645 (50.43)	696 (55.19)	830 (59.97)	1791 (60.47)	
BMI, kg/m^2^	24.19 ± 3.44	24.16 ± 3.55	24.09 ± 3.45	24.03 ± 3.34	0.455
Smoking status, *n* (%)					
Non−/ex-smoker	898 (70.21)	976 (77.4)	1,137 (82.15)	2,447 (82.61)	<0.001
Current smoker	381 (29.79)	285 (22.6)	247 (17.85)	515 (17.39)	
Alcohol consumption, *n* (%)					0.088
None	292 (22.83)	266 (21.09)	324 (23.41)	657 (22.18)	
≤ 1 drink/month	360 (28.15)	380 (30.13)	377 (27.24)	891 (30.08)	
2 drinks/month to 3 drinks/week	509 (39.8)	519 (41.16)	594 (42.92)	1,206 (40.72)	
≥ 4 drinks/week	118 (9.23)	96 (7.61)	89 (6.43)	208 (7.02)	
Educational level, *n* (%)					<0.001
Elementary school	147 (11.51)	90 (7.15)	89 (6.44)	210 (7.09)	
Middle school	190 (14.88)	127 (10.1)	147 (10.63)	268 (9.05)	
High school	538 (42.13)	477 (37.92)	571 (41.29)	1,134 (38.3)	
College or university	402 (31.48)	564 (44.83)	576 (41.65)	1,349 (45.56)	
Occupation, *n* (%)					<0.001
Unemployed (student, housewife, etc.)	205 (16.04)	286 (22.73)	424 (30.72)	793 (26.82)	
Office worker	320 (25.04)	424 (33.7)	409 (29.64)	971 (32.84)	
Sales and services	263 (20.58)	213 (16.93)	224 (16.23)	500 (16.91)	
Agriculture, forestry, fisheries	365 (28.56)	241 (19.16)	219 (15.87)	432 (14.61)	
Manual labor	125 (9.78)	94 (7.47)	104 (7.54)	261 (8.83)	
Household income, *n* (%)					<0.001
Low	140 (10.96)	108 (8.57)	118 (8.54)	255 (8.62)	
Low-moderate	309 (24.2)	269 (21.35)	291 (21.06)	594 (20.09)	
Moderate-high	425 (33.28)	365 (28.97)	410 (29.67)	909 (30.74)	
High	403 (31.56)	518 (41.11)	563 (40.74)	1,199 (40.55)	
Marital status, *n* (%)					0.037
Single	69 (5.56)	74 (6.03)	67 (4.94)	180 (6.18)	
Married	39 (3.14)	34 (2.77)	52 (3.83)	135 (4.64)	
Separated/divorced/widowed	1,133 (91.3)	1,119 (91.2)	1,237 (91.22)	2,597 (89.18)	
Sleep duration, hours	7.21 ± 1.25	7.15 ± 1.13	7.13 ± 1.2	7.07 ± 1.17	0.006
Walking duration per session, min	0	56.28 ± 63.28	53.58 ± 52.69	66.23 ± 60.98	<0.001
Weekly walking duration, min	0	144.2 ± 161.5	234.7 ± 236.9	491.1 ± 471.5	<0.001
Depressive mood, *n* (%)	60 (4.69)	37 (2.93)	38 (2.75)	93 (3.14)	0.020
Suicidal ideation, *n* (%)	29 (2.27)	17 (1.35)	13 (0.94)	23 (0.78)	<0.001
Comorbidities, *n* (%)					
Hypertension	265 (20.72)	218 (17.29)	229 (16.55)	521 (17.59)	0.027
Diabetes	87 (6.8)	83 (6.58)	103 (7.44)	202 (6.82)	0.830
Dyslipidemia	246 (19.23)	208 (16.49)	251 (18.14)	512 (17.29)	0.278
Stroke	24 (1.88)	14 (1.11)	18 (1.3)	38 (1.28)	0.350
Myocardial infarction	8 (0.63)	6 (0.48)	9 (0.65)	6 (0.2)	0.091
Angina	11 (0.86)	14 (1.11)	12 (0.87)	27 (0.91)	0.900
Malignancy	15 (1.17)	12 (0.95)	19 (1.37)	45 (1.52)	0.483

BMI, body mass index.

### Relationships of walking activity with depressed mood and suicidal ideation

**Figure 2:**
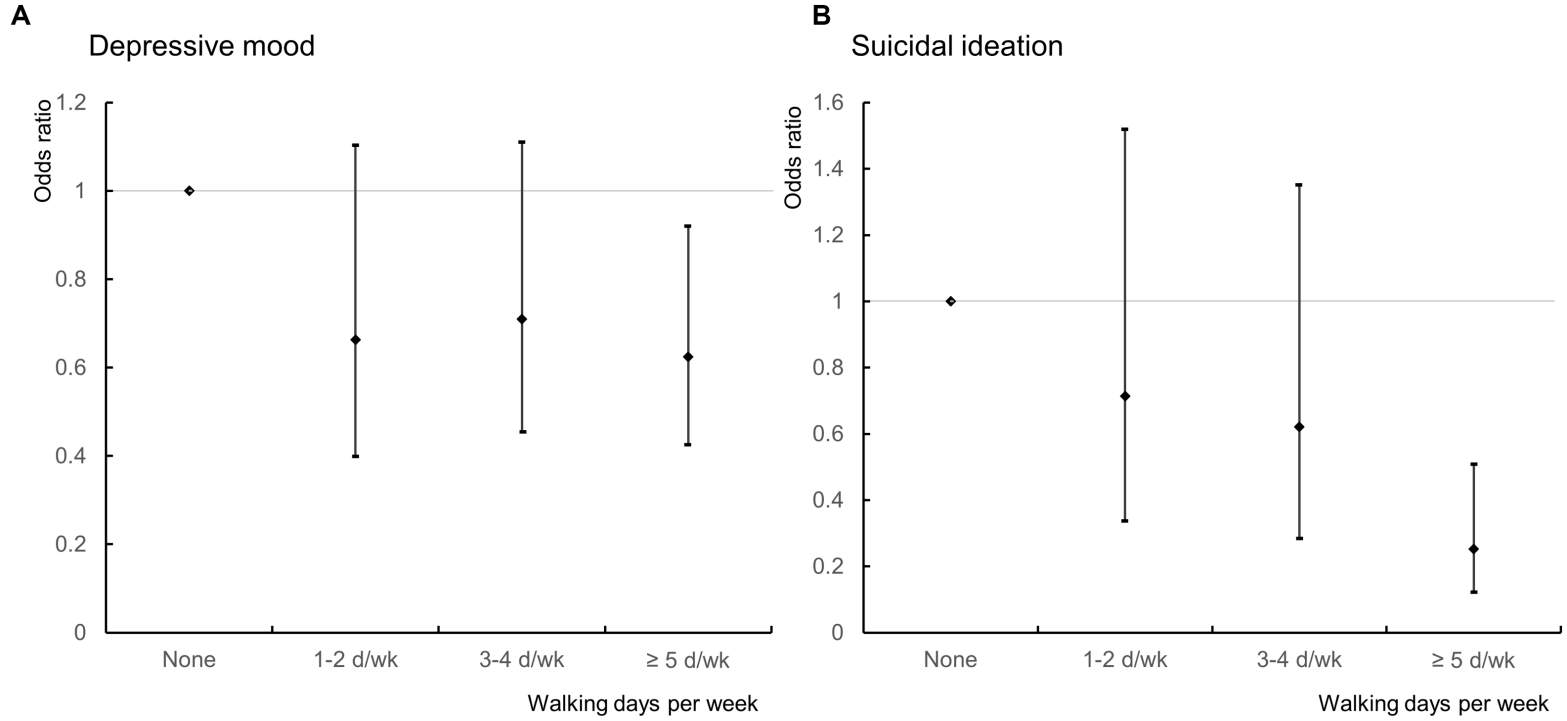
Associations of walking activity with **(A)** depressed mood and **(B)** suicidal ideation.

The estimated ORs for depressed mood and suicidal ideation derived from the multiple logistic regression analyzes are presented in Table 2. In the crude model, the ORs for depressed mood in the 1–2 days per week, 3–4 days per week, and ≥ 5 days per week groups were lower than the ORs of the group who never walked [0.608 (95% CI: 0.378–0.977, *p* = 0.04), 0.615 (95% CI: 0.4–0.946, *p* = 0.027), and 0.556 (95% CI: 0.393–0.786, *p* = 0.001), respectively]. Compared with the participants who never walked, those who walked 3–4 days per week and ≥ 5 days per week had lower ORs (0.483, 95% CI: 0.247–0.948, *p* = 0.034, and 0.335, 95% CI: 0.194–0.578, *p* < 0.001, respectively) for suicidal ideation. Walking duration per session and weekly walking duration were not associated with a depressed mood or suicidal ideation. In the adjusted model, participants who walked on ≥5 days per week had significantly lower ORs for depressed mood (0.625, 95% CI: 0.424–0.921, *p* = 0.018) and suicidal ideation (0.252, 95% CI: 0.125–0.507, *p* < 0.001) compared with participants who never walked (Figure 2). Walking duration per session and weekly walking duration were not associated with depressed mood or suicidal ideation.

**Table 2:** Relationships of walking with depressed mood and suicidal ideation.

	Crude model			Adjusted model*		
	OR	95% CI	*p*-value	OR	95% CI	*p*-value
Number of walking days						
None	1			1		
1–2 d/week	0.608	0.378–0.977	0.040	0.663	0.399–1.104	0.114
Walking duration per session, min	1.000	0.995–1.006	0.955	1.000	0.993–1.006	0.939
3–4 d/week	0.615	0.400–0.946	0.027	0.710	0.454–1.110	0.133
Walking duration per session, min	1.002	0.996–1.007	0.571	1.002	0.996–1.008	0.504
≥ 5 d/week	0.556	0.393–0.786	0.001	0.625	0.424–0.921	0.018
Walking duration per session, min	1.002	0.999–1.005	0.265	1.002	0.998–1.005	0.437
Weekly walking duration, min	0.999	0.999–1.000	0.828	1.000	0.999–1.001	0.883
Number of walking days						
None	1			1		
1–2 d/week	0.593	0.284–1.236	0.163	0.714	0.336–1.519	0.381
Walking duration per session, min	1.001	0.994–1.009	0.778	0.996	0.986–1.007	0.473
3–4 d/week	0.483	0.247–0.945	0.034	0.621	0.286–1.351	0.229
Walking duration per session, min	0.999	0.985–1.031	0.900	1.003	0.988–1.019	0.669
≥ 5 d/week	0.335	0.194–0.578	< 0.001	0.252	0.125–0.507	< 0.001
Walking duration per session, min	1.002	0.997–1.006	0.470	1.001	0.994–1.007	0.868
Weekly walking duration, min	0.999	0.997–1.001	0.118	0.999	0.998–1.000	0.151

OR, odds ratio; CI, confidence interval. *Adjusted for age, sex, smoking status, alcohol consumption, educational level, occupation, household income, marital status, sleep duration, and comorbidities.

### Associations of walking activity with depressed mood and suicidal ideation stratified by sex

We performed a stratified analysis to explore the possible effects of sex. Male participants who walked on ≥5 days per week had a lower risk of a depressed mood (OR = 0.625, 95% CI: 0.424–0.921, *p* = 0.018) and suicidal ideation (OR = 0.252, 95% CI: 0.125–0.507, *p* < 0.001) compared with participants who never walked. Female participants walking on >5 days per week had a significantly higher risk of suicidal ideation (OR = 0.269, 95% CI: 0.106–0.667, *p* = 0.005; Table 3). In a supplementary analysis conducted on 30 min of walking, walking 5 or more times a week was also associated with a lower risk of suicidal ideation (OR 0.315, 95% CI 0.157–0.633; Supplementary Table S2).

**Table 3:** Associations of walking with depressed mood and suicidal ideation stratified by sex.

		Depressive mood			Suicidal ideation		
		OR	95% CI	*p*-value	OR	95% CI	*p*-value
Male	None	1			1		
	1–2 d/week	0.663	0.399–1.104	0.114	0.714	0.336–1.519	0.381
	3–4 d/week	0.710	0.454–1.110	0.133	0.621	0.286–1.351	0.229
	≥ 5 d/week	0.625	0.424–0.921	0.018	0.252	0.125–0.507	<0.001
Female	None	1			1		
	1–2 d/week	0.880	0.436–1.775	0.720	0.994	0.437–2.258	0.988
	3–4 d/week	0.900	0.493–1.643	0.731	0.674	0.250–1.821	0.436
	≥ 5 d/week	0.704	0.406–1.221	0.211	0.269	0.106–0.667	0.005

OR, odds ratio; CI, confidence interval. Adjusted for age, smoking status, alcohol consumption, educational level, occupation, household income, marital status, sleep duration, and comorbidities.

## Discussion

The results of this nationwide cross-sectional study of 6,886 middle-aged adults in South Korea suggest a beneficial effect of walking on depressed mood and suicidal ideation in this population. Regularly walking (> 5 times per week) was associated with a 40% reduction in depressed mood and a > 70% reduction in suicidal ideation compared to not walking at all among middle-aged adults. Suicidal ideation decreased with a greater number of walking days, though not significant for 1–2 days/week and 3–4 days/week. Depressive mood showed a numerical decrease for 1–2 days/week and 3–4 days/week, although it was not statistically significant. These effects were observed after adjusting for various factors such as age, smoking status, alcohol consumption, education level, occupation, household income, marital status, sleep duration, and comorbidities. Interestingly, the duration of a single exercise occasion and the total exercise time during the week did not modulate the results. In other words, exercising for a long period on one occasion did not have a beneficial effect; benefits were only seen when regularly exercising (> 5 times per week). Thus, it is important walk almost every day, even for a short time. Sex-stratified analysis revealed similar findings for depressed mood and suicidal ideation in men, but only suicidal ideation showed significant association in women.

Our results share some similarities with previous studies on the association between walking and mental health. A study of older females reported that walking for 50 min, 3 times per week, helped reduce depression (21). A meta-analysis of eight randomized controlled trials reported a clinical effect size of −0.86 for walking to treat depression (22). Exercise increases central arousal and releases various neurotransmitters, such as dopamine, epinephrine, norepinephrine, serotonin, and endorphins (23–25). This release of neurotransmitters can improve mood, emotional functioning, and stress reactivity (26). Exercise also upregulates growth factors, such as brain-derived neurotrophic factor, which is associated with depression and suicidal ideation (26–28).

To understand the need for suicide prevention measures in middle-aged adults, it is important to examine the recent rise in suicide rates in this group, including in terms of the potential impact on society. The Great Recession, which occurred in the United States from 2007 to 2009, had a global economic impact, and also affected the mental health of economically active middle-aged individuals (3). Additionally, the rapid growth of South Korea since the 1998 financial crisis, which was aided by the International Monetary Fund, has imposed economic and employment pressure on young and middle-aged individuals (29, 30). Even without these issues, middle-aged individuals often face pressure to achieve in various aspects of life. In Erikson’s original psychosocial stages, middle age is characterized as either generative or stagnated, and recent studies have emphasized the importance of engaging in generative activities during this life stage (31). Middle-aged individuals play important roles in society, contributing to the workplace, parenting, and various community activities (32). Hence, the rising suicide rate among this population is a grave concern; it could potentially impose a major burden on society, thus making it a public health issue.

Previous studies have identified suicidal ideation and depression as significant risk factors for suicide among middle-aged adults (8, 33). Although pharmacotherapy is considered the primary treatment in most cases, non-pharmacological therapy is often used concurrently, or even in preference to pharmacotherapy (due to side effects, poor compliance, and the patient’s health status) (34). Exercise has been proposed as an alternative therapy for depression and suicidal ideation (9, 10). In a previous study, 30 min of walking, 4 times a week for 4 weeks helped to increase the patients’ adaptive coping strategies (15). In line with this, our supplementary analysis also showed that walking of >30 min for more than 5 times a week was associated with a decrease in suicidal ideation. However, an issue with exercise therapy is that patients who are depressed or have suicidal ideation often lack motivation, and the idea of having to start to exercise in earnest can be daunting. Therefore, it is more interesting and encouraging that significant results were obtained with only a short time of exercise of more than 10 min, as in our study. We hope that our results highlight the usefulness of walking and help patients become motivated. Walking is an exercise that requires no equipment and can be easily started in the neighborhood or during commuting hours, thus making it relatively accessible (35). Our findings suggest that regular walking, regardless of duration, has a beneficial effect on middle-aged individuals, and may be especially helpful for those who have various responsibilities at work and home. Walking is a simple way to maintain mental health.

As this study was based on national health data, several points should be considered. First, because the data were not collected specifically for this study, it was difficult to apply data collection strategies commonly used in research (36). Moreover, as this was a cross-sectional study, it was not possible to identify causal relationships. Our interpretation of the results may have been reversed; it could also be that individuals without mood symptoms could walk more. Also, the data on exercise, depressed mood, and suicidal ideation relied on self-report measures; thus, recall bias could have affected the results. Finally, the family type was not included in the adjusted variables, because of the concern of possible multicollinearity with the marital status.

To the best of our knowledge, this is the first study to investigate the associations of walking with depressed mood and suicidal ideation in middle-aged adults. Regardless of the duration of exercise, walking >5 times per week helped reduce depressed mood and suicidal ideation in middle-aged men, and suicidal ideation in middle-aged women. Clinicians could recommend walking as a non-pharmacological approach to reduce patient suicidal ideation. Short walks could serve as a useful starting point for patients with serious mental health issues, such as depressed mood and/or suicidal ideation.

## Conclusion

In this nationwide cross-sectional study of middle-aged adults in South Korea, walking  >5 times per week was helpful for mental health, regardless of duration. The study found an association between walking and reduced symptoms of depressed mood and suicidal ideation in middle-aged men, and a significant reduction in suicidal ideation in middle-aged women.

## Data availability statement

The original contributions presented in the study are included in the article/Supplementary material, further inquiries can be directed to the corresponding author.

## Ethics statement

The studies involving humans were approved by the Institutional Review Board of the KCDC (Korea Disease Control and Prevention Agency) approved the KNHANES VII (approval no. 2018-01-03-P-A). The studies were conducted in accordance with the local legislation and institutional requirements. The participants provided their written informed consent to participate in this study.

## Author contributions

YK and YC contributed to data analysis and writing the manuscript. GK, CH, SS, and HR gave critical opinions on the study design, and the manuscript. JM contributed to data analysis. SH contributed to interpreting the results and supervised the entire process. All authors contributed to the literature review, study design, data interpretation, and approved the final manuscript.

## Funding

This work was supported by the Soonchunhyang University Research Fund (2023–0018). The Korea Health Technology R&D Project through the Korea Health Industry Development Institute (KHIDI) funded by the Ministry of Health &Welfare, Republic of Korea (grant number: HR22C173401).

## Conflict of interest

The authors declare that the research was conducted in the absence of any commercial or financial relationships that could be construed as a potential conflict of interest.

## Publisher’s note

All claims expressed in this article are solely those of the authors and do not necessarily represent those of their affiliated organizations, or those of the publisher, the editors and the reviewers. Any product that may be evaluated in this article, or claim that may be made by its manufacturer, is not guaranteed or endorsed by the publisher.
