# Supplementary material for: Relationships of walking activity with depressed mood and suicidal ideation among the middle-aged Korean population: a nationwide cross-sectional study
Source: Front Psychiatry. 2023 Sep 5;14:1202068. doi: 10.3389/fpsyt.2023.1202068 (PMC10512318; doi:10.3389/fpsyt.2023.1202068)
Supplement: Supplementary file 1 [file Table_1.docx]

**Supplementary Table 1. Baseline characteristics according to the number of walking days per week (weighting applied).**

|  | **None (n = 1,525,642)** | | **1-2 d/wk (n = 1,513,985)** | | **3-4 d/wk (n = 1,680,730)** | | **≥ 5 d/wk (n = 3,580,193)** | | **P-value** |
| --- | --- | --- | --- | --- | --- | --- | --- | --- | --- |
|  | **Mean or n (%)** | **SE** | **Mean or n (%)** | **SE** | **Mean or n (%)** | **SE** | **Mean or n (%)** | **SE** |  |
| Age, years | 50.15 | 0.21 | 49.64 | 0.20 | 49.94 | 0.18 | 49.93 | 0.13 | 0.289 |
| Sex, n (%) |  |  |  |  |  |  |  |  | <0.001 |
| Male | 873848 (57.28) | 41043.14 | 785178 (51.86) | 42771.55 | 781780 (46.51) | 38438.07 | 1681684 (46.97) | 59421.36 |  |
| Female | 651794 (42.72) | 31281.19 | 728807 (48.14) | 33380.87 | 898950 (53.49) | 38577.15 | 1898510 (53.03) | 57621.87 |  |
| BMI, kg/m^2^ | 24.24 | 0.11 | 24.26 | 0.10 | 24.09 | 0.10 | 24.12 | 0.07 | 0.472 |
| Smoking status, n (%) |  |  |  |  |  |  |  |  |  |
| Non-/ex-smoker | 1022118 (67.00) | 42891.7 | 1125625 (74.35) | 50912.71 | 1335216 (79.44) | 53997.45 | 2870327 (80.17) | 79883.08 | <0.001 |
| Current smoker | 503524 (33.00) | 31599.31 | 388360 (25.65) | 28180.65 | 345514 (20.56) | 24456.35 | 709867 (19.83) | 36024.59 |  |
| Alcohol consumption, n (%) |  |  |  |  |  |  |  |  | 0.270 |
| None | 332034 (21.76) | 22179.42 | 284988 (18.82) | 21082.6 | 371124 (22.08) | 23427.69 | 735521 (20.54) | 34358.51 |  |
| ≤ 1 drink/month | 413739 (27.12) | 24934.38 | 441709 (29.18) | 27303.61 | 443895 (26.41) | 27538.27 | 1041301 (29.09) | 44717.33 |  |
| 2 drinks/month to 3 drinks/week | 634677 (41.60) | 34741.25 | 663314 (43.81) | 38484.39 | 747827 (44.49) | 38078.96 | 1528892 (42.70) | 54796.11 |  |
| ≥ 4 drinks/week | 145193 (9.52) | 15809.28 | 123973 (8.19) | 14616.37 | 117883 (7.02) | 14024.28 | 274479 (7.67) | 22573.12 |  |
| Educational level, n (%) |  |  |  |  |  |  |  |  | <0.001 |
| Elementary school | 162068 (10.64) | 16259.72 | 91165 (6.04) | 11445.73 | 92631 (5.51) | 11377.3 | 227793 (6.36) | 18375.06 |  |
| Middle school | 222697 (14.62) | 18865.93 | 154101 (10.20) | 17157.07 | 177183 (10.55) | 17659.1 | 300369 (8.39) | 20466.51 |  |
| High school | 648999 (42.61) | 36825.01 | 566772 (37.52) | 30861.7 | 699053 (41.61) | 35513.48 | 1402749 (39.19) | 54632.64 |  |
| College or university | 489322 (32.13) | 30441.22 | 698542 (46.24) | 42659.58 | 710981 (42.32) | 42193.84 | 1648479 (46.05) | 67441.66 |  |
| Occupation, n (%) |  |  |  |  |  |  |  |  | <0.001 |
| Unemployed (student, housewife, etc.) | 216246 (14.18) | 18246.59 | 309431 (20.50) | 21479 | 500485 (29.85) | 30637.06 | 889338 (24.89) | 37252.35 |  |
| Office worker | 400300 (26.26) | 30260.6 | 516295 (34.21) | 33852.15 | 500756 (29.86) | 31156.09 | 1190470 (33.32) | 54354.63 |  |
| Sales and services | 308773 (20.25) | 23120.41 | 255838 (16.95) | 22054.59 | 261720 (15.61) | 21777.82 | 599141 (16.77) | 32044.4 |  |
| Agriculture, forestry, fisheries | 464988 (30.50) | 29434.11 | 317564 (20.04) | 24887.83 | 294188 (17.54) | 23371.49 | 562117 (15.73) | 34521.65 |  |
| Manual labor | 134290 (8.81) | 13090.04 | 110224 (7.30) | 13062.07 | 119679 (7.14) | 13416.17 | 332006 (9.29) | 24704.54 |  |
| Household income, n (%) |  |  |  |  |  |  |  |  | <0.001 |
| Low | 168510 (11.07) | 19234.34 | 125836 (8.32) | 14119.59 | 131953 (7.87) | 14533 | 302777 (8.47) | 23180.88 |  |
| Low-moderate | 353783 (23.24) | 25455.73 | 309091 (20.43) | 22172.32 | 377169 (22.48) | 26191.9 | 691929 (19.37) | 35582.88 |  |
| Moderate-high | 523835 (34.41) | 31465.02 | 446669 (29.52) | 29469.36 | 488339 (29.11) | 29172.23 | 1124358 (31.47) | 52061 |  |
| High | 476342 (31.29) | 29802.66 | 631414 (41.73) | 40520.29 | 680180 (40.54) | 42393.47 | 1453615 (40.69) | 66895.66 |  |
| Marital status, n (%) |  |  |  |  |  |  |  |  | 0.052 |
| Single | 88264 (5.94) | 13041.21 | 88752 (6.01) | 11032.84 | 91489 (5.56) | 12991.25 | 226404 (6.42) | 19371.83 |  |
| Married | 38215 (2.57) | 6552.15 | 43490 (2.95) | 8717.23 | 59382 (3.61) | 9584.86 | 166820 (4.73) | 16768.99 |  |
| Separated/divorced/widowed | 1359700 (91.49) | 52081.2 | 1343566 (91.04) | 55630.85 | 1494237 (90.83) | 56424.45 | 3134430 (88.85) | 87832.01 |  |
| Sleep duration, hours | 7.18 | 0.04 | 7.16 | 0.03 | 7.14 | 0.04 | 7.08 | 0.02 | 0.098 |
| Walking duration per session, min | 0 | - | 56.71 | 2.27 | 52.59 | 1.62 | 64.73 | 1.18 | <0.001 |
| Weekly walking duration, min | 0 | - | 145.32 | 6.15 | 230.22 | 7.21 | 479.22 | 8.92 | <0.001 |
| Depressive mood, n (%) | 73497 (4.82) | 11236 | 45165 (2.98) | 8768.28 | 50759 (3.02) | 9724.2 | 97582 (2.73) | 12431.35 | 0.012 |
| Suicidal ideation, n (%) | 34117 (2.24) | 7399.48 | 20260 (1.34) | 6457.98 | 18371 (1.09) | 5716.28 | 20864 (0.58) | 4926.94 | <0.001 |
| Comorbidities, n (%) |  |  |  |  |  |  |  |  |  |
| Hypertension | 312587 (20.49) | 23351.63 | 257573 (17.01) | 19452.1 | 268805 (15.99) | 20882.98 | 637515 (17.81) | 32692.94 | 0.052 |
| Diabetes | 104311 (6.84) | 13789.13 | 97889 (6.47) | 12677.1 | 122238 (7.27) | 13819.55 | 241719 (6.75) | 19520.69 | 0.910 |
| Dyslipidemia | 293376 (19.23) | 22480.62 | 245939 (16.24) | 20118.21 | 280653 (16.70) | 20505.88 | 623817 (17.42) | 32712.29 | 0.300 |
| Stroke | 27686 (1.81) | 7047.32 | 13603 (0.90) | 3880.17 | 22698 (1.35) | 7015.94 | 48022 (1.34) | 8711.15 | 0.400 |
| Myocardial infarction | 9136 (0.60) | 3870.34 | 7782 (0.51) | 3562.99 | 10996 (0.65) | 3841.85 | 6211 (0.17) | 2702.89 | 0.100 |
| Angina | 9553 (0.63) | 3050.92 | 18158 (1.20) | 5823.91 | 12661 (0.75) | 4682.51 | 32323 (0.90) | 7540.25 | 0.550 |
| Malignancy | 15792 (1.04) | 4476.63 | 12994 (0.86) | 4328.73 | 19639 (1.17) | 4899.30 | 49175 (1.37) | 8239.98 | 0.530 |
| Continuous variables are presented as weighted mean and categorical variables are presented as n (weighted percentage). SE, standard error; BMI, body mass index | | | | | | | | | |

**Supplementary Table 2. Relationships of walking more than 30 minutes with depressed mood and suicidal ideation**

|  | **Depressive mood** | | |  | **Suicidal ideation** | | |
| --- | --- | --- | --- | --- | --- | --- | --- |
|  | **OR** | **95% CI** | **P-value** |  | **OR** | **95% CI** | **P-value** |
| Number of walking days (≥ 30 min) |  |  |  |  |  |  |  |
| None | 1 |  |  |  | 1 |  |  |
| 1–2 d/week | 0.802 | 0.458–1.405 | 0.440 |  | 0.713 | 0.293–1.737 | 0.456 |
| 3–4 d/week | 0.913 | 0.572–1.457 | 0.702 |  | 0.606 | 0.270–1.360 | 0.224 |
| ≥ 5 d/week | 0.795 | 0.554–1.139 | 0.211 |  | 0.315 | 0.157–0.633 | <0.001 |
| OR, odds ratio; CI, confidence interval.  Adjusted for age, smoking status, alcohol consumption, educational level, occupation, household income, marital status, sleep duration, and comorbidities | | | | | | | |
